# Retraction Note: The effects of dexamethasone on the proliferation and apoptosis of human ovarian cancer cells induced by paclitaxel

**DOI:** 10.1186/s13048-015-0144-4

**Published:** 2015-03-26

**Authors:** Wenjing Hou, Jianhua Guan, Huan Lu, Qian Dong, Yuhong Han, Rong Zhang

**Affiliations:** Department of Obstetrics and Gynecology, Shanghai Jiaotong University-Affiliated Sixth People’s Hospital of Fengxian Branch, 6600 Nanfeng Road, Shanghai, 201499 People’s Republic of China

## Retraction

The Publisher and Editor regretfully retract this article [1] because the peer-review process was inappropriately influenced and compromised. As a result, the scientific integrity of the article cannot be guaranteed. A systematic and detailed investigation suggests that a third party was involved in supplying fabricated details of potential peer reviewers for a large number of manuscripts submitted to different journals. In accordance with recommendations from COPE we have retracted all affected published articles, including this one. It was not possible to determine beyond doubt that the authors of this particular article were aware of any third party attempts to manipulate peer review of their manuscript.
